# Treatment of Bed-sores

**Published:** 1845-01

**Authors:** 


*Treatment of Bed-sores.*—A writer, in a recent number of *Walther* and *Ammon's* Journal, recommends the application of a lotion composed of equal parts of spirits of camphor and the vegeto-mineral water of Goulard. The parts, that have become red by the pressure, should be repeatedly moistened with this lotion; it requires to be briskly shaken before it is applied.

If, in spite of this treatment, the skin should break, the zinc or lead ointment, to which some camphor has been added, is a good application. In still more obstinate cases, an ointment, consisting of four parts of fresh-prepared tannate of lead, and thirty of lard, has been sometimes found to answer extremely well. On the whole, however, nothing succeeds so uniformly, alike as a prophylactic remedy against the abrasion of the skin and a healing one to that which has become broken, as a solution of creosote—prepared after the method of Reichenbach—in the proportion of one part of the oil to 80 parts of water.

When the affected part becomes gangrenous, fomentations with a decoction of yellow bark, to which some tincture of myrrh has been added, may be useful. Some patients have found benefit from the sprinkling of the ulcerated surface with a powder composed of bark, camphor, and myrrh; others, from the use of the camphorated styrax ointment. The tinct. Benzoini compos., or Friar's balsam, is often an excellent application to bed-sores. Whatever be the nature of the application employed, the most important remedy of all is the removal of pressure from the affected parts, by means of air or water cushions. The comfort derived from the use of these is most pleasing.—*Ibid.*

